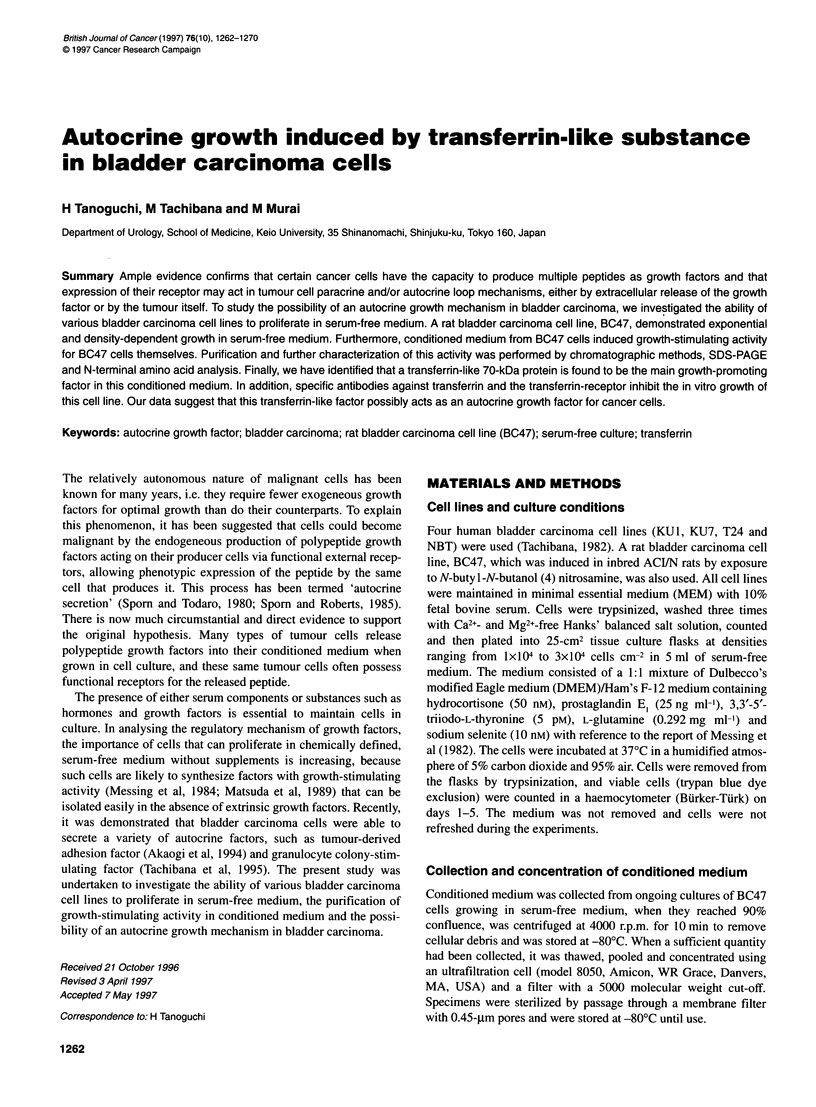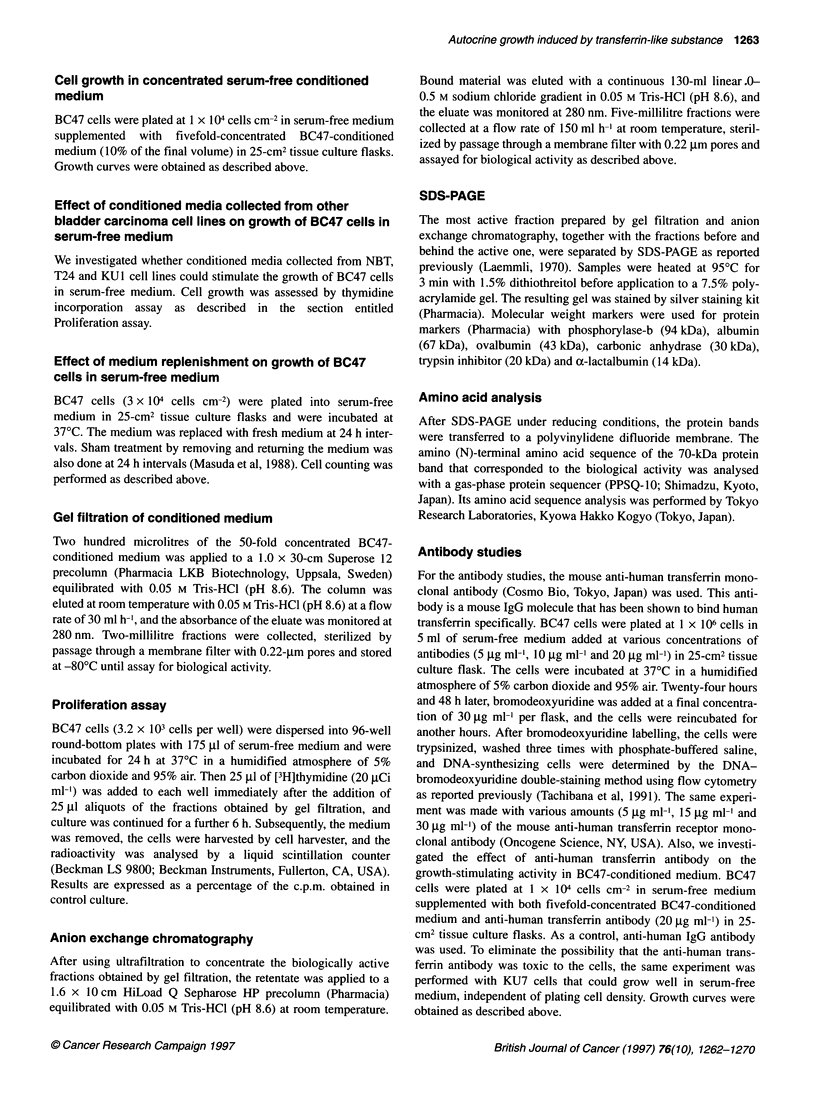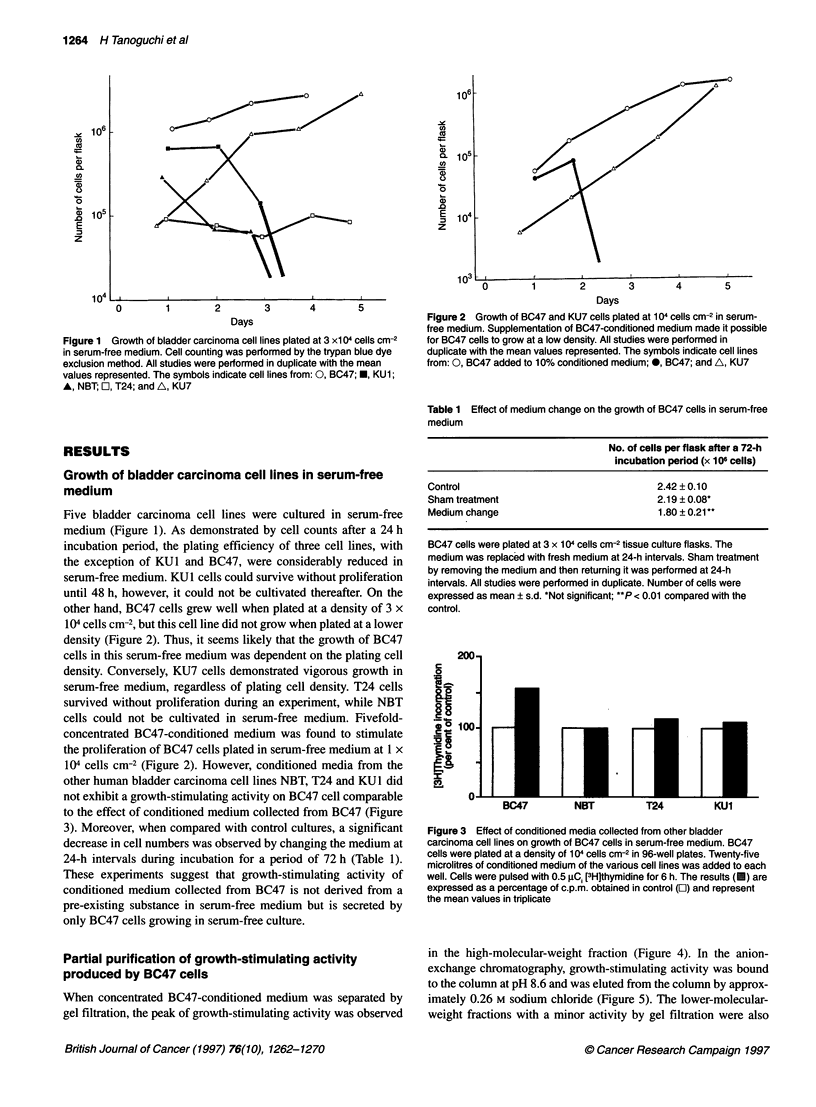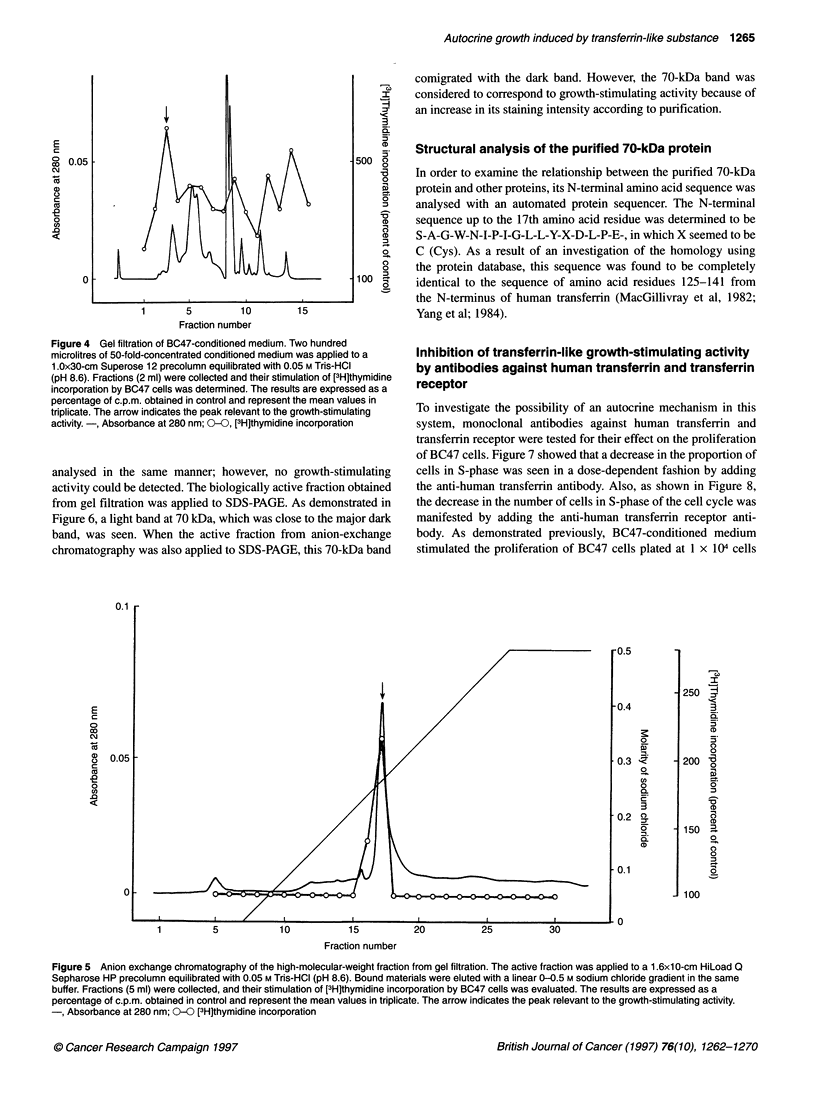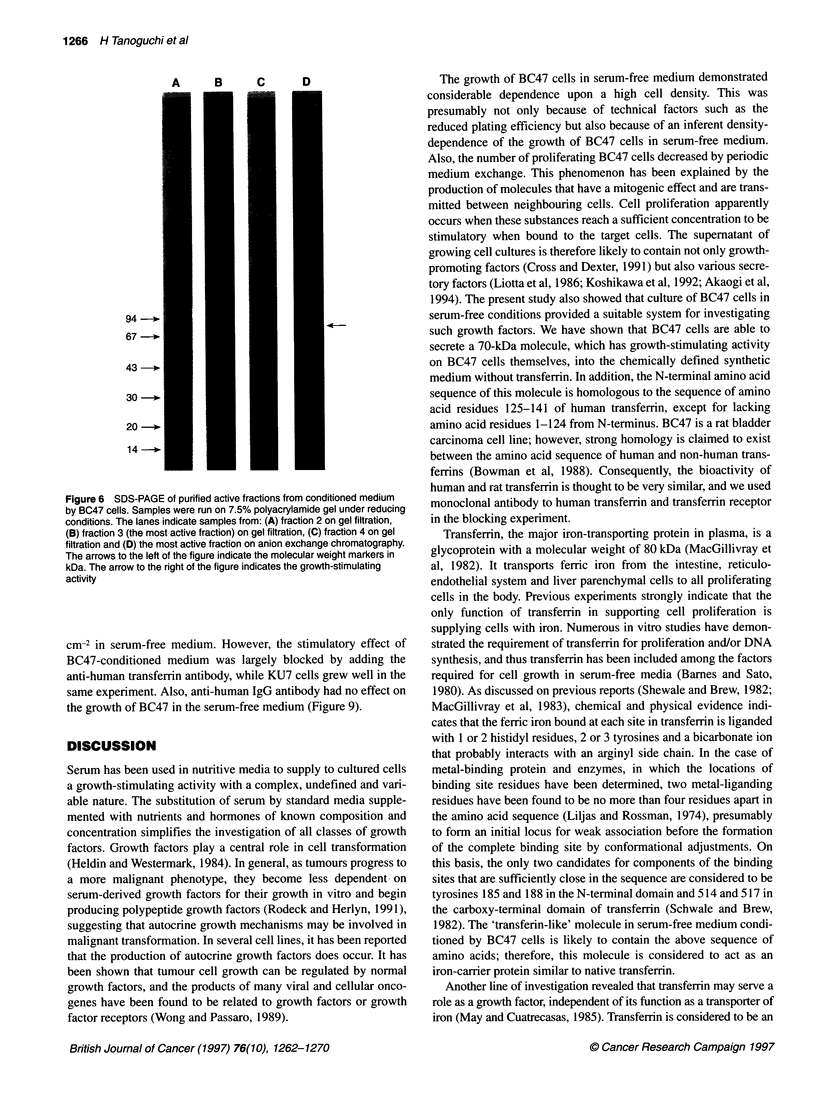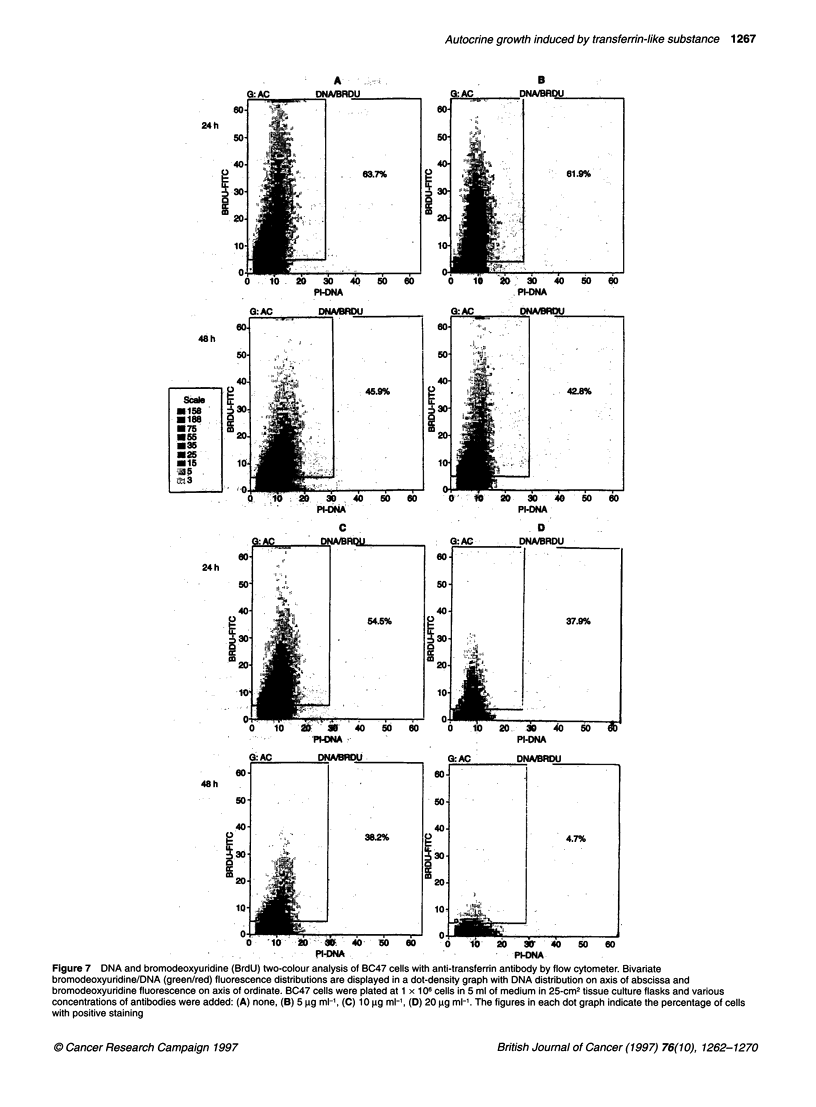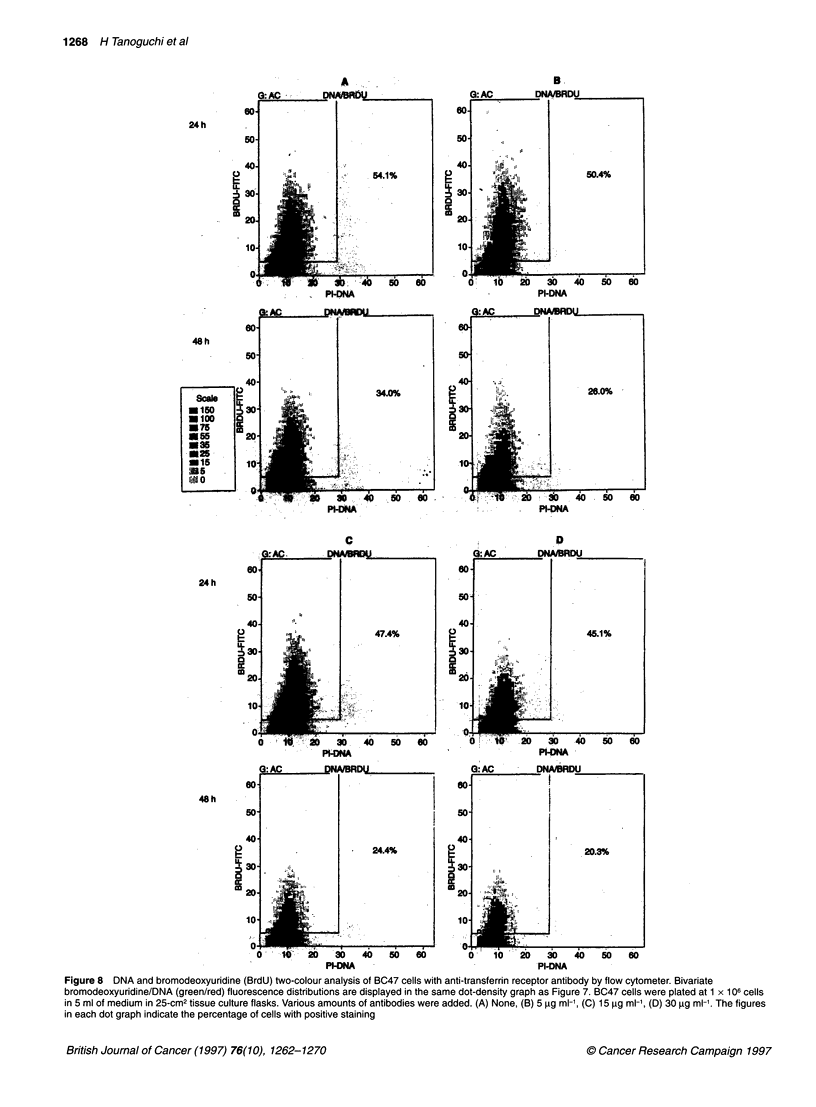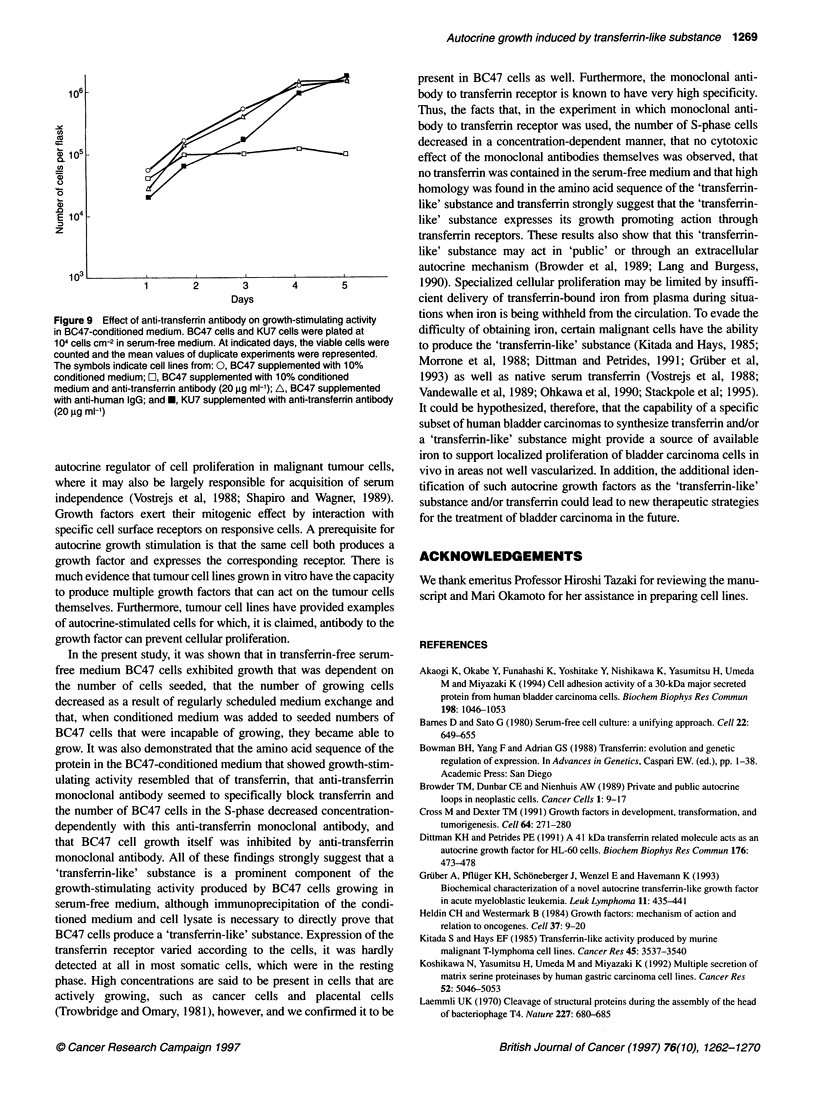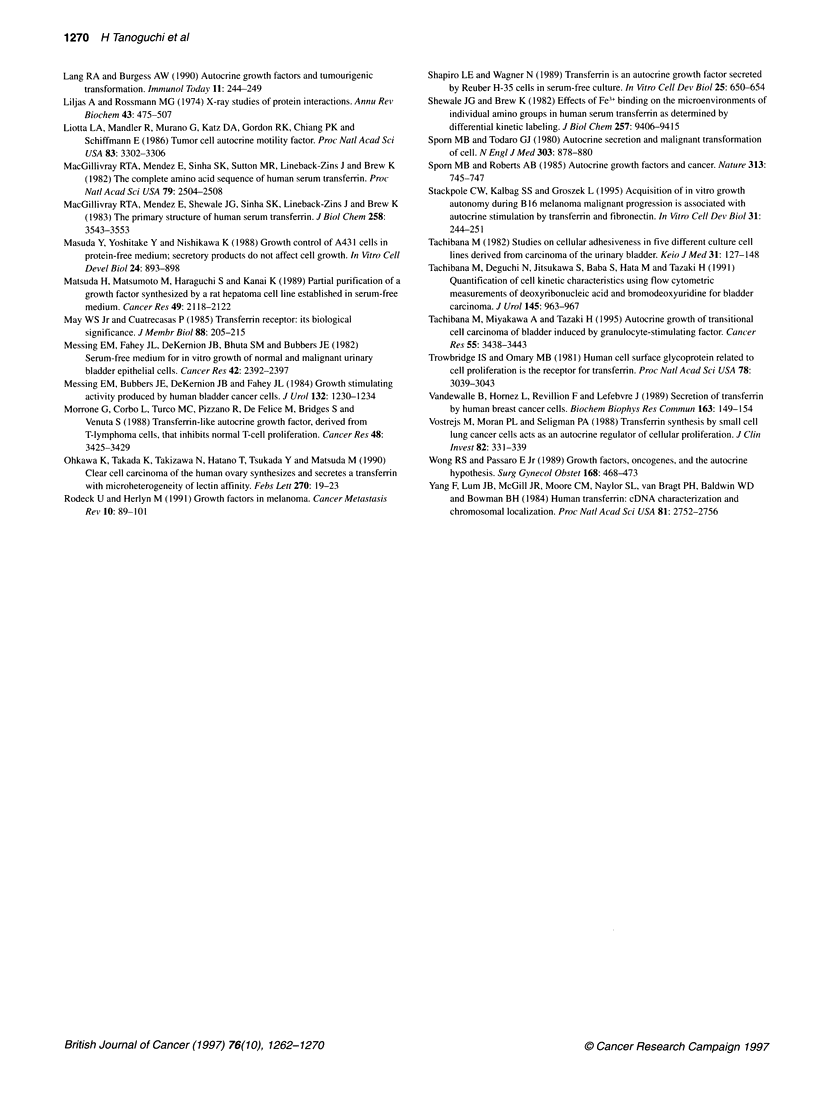# Autocrine growth induced by transferrin-like substance in bladder carcinoma cells.

**DOI:** 10.1038/bjc.1997.546

**Published:** 1997

**Authors:** H. Tanoguchi, M. Tachibana, M. Murai

**Affiliations:** Department of Urology, School of Medicine, Keio University, Tokyo, Japan.

## Abstract

**Images:**


*British Journal of Cancer* (1997) **76**(10), 1262–1270
© 1997 Cancer Research Campaign

### Autocrine growth induced by transferrin-like substance in bladder carcinoma cells

**H Tanoguchi, M Tachibana and M Murai**

Department of Urology, School of Medicine, Keio University, 35 Shinanomachi, Shinjuku-ku, Tokyo 160, Japan

**Summary** Ample evidence confirms that certain cancer cells have the capacity to produce multiple peptides as growth factors and that expression of their receptor may act in tumour cell paracrine and/or autocrine loop mechanisms, either by extracellular release of the growth factor or by the tumour itself. To study the possibility of an autocrine growth mechanism in bladder carcinoma, we investigated the ability of various bladder carcinoma cell lines to proliferate in serum-free medium. A rat bladder carcinoma cell line, BC47, demonstrated exponential and density-dependent growth in serum-free medium. Furthermore, conditioned medium from BC47 cells induced growth-stimulating activity for BC47 cells themselves. Purification and further characterization of this activity was performed by chromatographic methods, SDS-PAGE and N-terminal amino acid analysis. Finally, we have identified that a transferrin-like 70-kDa protein is found to be the main growth-promoting factor in this conditioned medium. In addition, specific antibodies against transferrin and the transferrin-receptor inhibit the in vitro growth of this cell line. Our data suggest that this transferrin-like factor possibly acts as an autocrine growth factor for cancer cells.

**Keywords:** autocrine growth factor; bladder carcinoma; rat bladder carcinoma cell line (BC47); serum-free culture; transferrin

The relatively autonomous nature of malignant cells has been known for many years, i.e. they require fewer exogeneous growth factors for optimal growth than do their counterparts. To explain this phenomenon, it has been suggested that cells could become malignant by the endogeneous production of polypeptide growth factors acting on their producer cells via functional external receptors, allowing phenotypic expression of the peptide by the same cell that produces it. This process has been termed 'autocrine secretion' (Sporn and Todaro, 1980; Sporn and Roberts, 1985). There is now much circumstantial and direct evidence to support the original hypothesis. Many types of tumour cells release polypeptide growth factors into their conditioned medium when grown in cell culture, and these same tumour cells often possess functional receptors for the released peptide.

The presence of either serum components or substances such as hormones and growth factors is essential to maintain cells in culture. In analysing the regulatory mechanism of growth factors, the importance of cells that can proliferate in chemically defined, serum-free medium without supplements is increasing, because such cells are likely to synthesize factors with growth-stimulating activity (Messing et al, 1984; Matsuda et al, 1989) that can be isolated easily in the absence of extrinsic growth factors. Recently, it was demonstrated that bladder carcinoma cells were able to secrete a variety of autocrine factors, such as tumour-derived adhesion factor (Akaogi et al, 1994) and granulocyte colony-stimulating factor (Tachibana et al, 1995). The present study was undertaken to investigate the ability of various bladder carcinoma cell lines to proliferate in serum-free medium, the purification of growth-stimulating activity in conditioned medium and the possibility of an autocrine growth mechanism in bladder carcinoma.

*Received 21 October 1996*
*Revised 3 April 1997*
*Accepted 7 May 1997*

*Correspondence to:* H Tanoguchi

### MATERIALS AND METHODS

### Cell lines and culture conditions

Four human bladder carcinoma cell lines (KU1, KU7, T24 and NBT) were used (Tachibana, 1982). A rat bladder carcinoma cell line, BC47, which was induced in inbred ACI/N rats by exposure to *N*-butyl-*N*-butanol (4) nitrosamine, was also used. All cell lines were maintained in minimal essential medium (MEM) with 10% fetal bovine serum. Cells were trypsinized, washed three times with $Ca^{2+}$- and $Mg^{2+}$-free Hanks' balanced salt solution, counted and then plated into 25-cm$^2$ tissue culture flasks at densities ranging from $1\times10^4$ to $3\times10^4$ cells cm$^{-2}$ in 5 ml of serum-free medium. The medium consisted of a 1:1 mixture of Dulbecco's modified Eagle medium (DMEM)/Ham's F-12 medium containing hydrocortisone (50 nM), prostaglandin $E_1$ (25 ng ml$^{-1}$), 3,3′-5′-triiodo-L-thyronine (5 pM), L-glutamine (0.292 mg ml$^{-1}$) and sodium selenite (10 nM) with reference to the report of Messing et al (1982). The cells were incubated at 37°C in a humidified atmosphere of 5% carbon dioxide and 95% air. Cells were removed from the flasks by trypsinization, and viable cells (trypan blue dye exclusion) were counted in a haemocytometer (Bürker-Türk) on days 1–5. The medium was not removed and cells were not refreshed during the experiments.

### Collection and concentration of conditioned medium

Conditioned medium was collected from ongoing cultures of BC47 cells growing in serum-free medium, when they reached 90% confluence, was centrifuged at 4000 r.p.m. for 10 min to remove cellular debris and was stored at –80°C. When a sufficient quantity had been collected, it was thawed, pooled and concentrated using an ultrafiltration cell (model 8050, Amicon, WR Grace, Danvers, MA, USA) and a filter with a 5000 molecular weight cut-off. Specimens were sterilized by passage through a membrane filter with 0.45-μm pores and were stored at –80°C until use.

### Cell growth in concentrated serum-free conditioned medium

BC47 cells were plated at $1 \times 10^4$ cells $cm^{-2}$ in serum-free medium supplemented with fivefold-concentrated BC47-conditioned medium (10% of the final volume) in 25-$cm^2$ tissue culture flasks. Growth curves were obtained as described above.

### Effect of conditioned media collected from other bladder carcinoma cell lines on growth of BC47 cells in serum-free medium

We investigated whether conditioned media collected from NBT, T24 and KU1 cell lines could stimulate the growth of BC47 cells in serum-free medium. Cell growth was assessed by thymidine incorporation assay as described in the section entitled Proliferation assay.

### Effect of medium replenishment on growth of BC47 cells in serum-free medium

BC47 cells ($3 \times 10^4$ cells $cm^{-2}$) were plated into serum-free medium in 25-$cm^2$ tissue culture flasks and were incubated at 37°C. The medium was replaced with fresh medium at 24 h intervals. Sham treatment by removing and returning the medium was also done at 24 h intervals (Masuda et al, 1988). Cell counting was performed as described above.

### Gel filtration of conditioned medium

Two hundred microlitres of the 50-fold concentrated BC47-conditioned medium was applied to a 1.0 × 30-cm Superose 12 precolumn (Pharmacia LKB Biotechnology, Uppsala, Sweden) equilibrated with 0.05 M Tris-HCl (pH 8.6). The column was eluted at room temperature with 0.05 M Tris-HCl (pH 8.6) at a flow rate of 30 ml $h^{-1}$, and the absorbance of the eluate was monitored at 280 nm. Two-millilitre fractions were collected, sterilized by passage through a membrane filter with 0.22-μm pores and stored at –80°C until assay for biological activity.

### Proliferation assay

BC47 cells ($3.2 \times 10^3$ cells per well) were dispersed into 96-well round-bottom plates with 175 μl of serum-free medium and were incubated for 24 h at 37°C in a humidified atmosphere of 5% carbon dioxide and 95% air. Then 25 μl of [$^3$H]thymidine (20 μCi $ml^{-1}$) was added to each well immediately after the addition of 25 μl aliquots of the fractions obtained by gel filtration, and culture was continued for a further 6 h. Subsequently, the medium was removed, the cells were harvested by cell harvester, and the radioactivity was analysed by a liquid scintillation counter (Beckman LS 9800; Beckman Instruments, Fullerton, CA, USA). Results are expressed as a percentage of the c.p.m. obtained in control culture.

### Anion exchange chromatography

After using ultrafiltration to concentrate the biologically active fractions obtained by gel filtration, the retentate was applied to a 1.6 × 10 cm HiLoad Q Sepharose HP precolumn (Pharmacia) equilibrated with 0.05 M Tris-HCl (pH 8.6) at room temperature. Bound material was eluted with a continuous 130-ml linear .0–0.5 M sodium chloride gradient in 0.05 M Tris-HCl (pH 8.6), and the eluate was monitored at 280 nm. Five-millilitre fractions were collected at a flow rate of 150 ml $h^{-1}$ at room temperature, sterilized by passage through a membrane filter with 0.22 μm pores and assayed for biological activity as described above.

### SDS-PAGE

The most active fraction prepared by gel filtration and anion exchange chromatography, together with the fractions before and behind the active one, were separated by SDS-PAGE as reported previously (Laemmli, 1970). Samples were heated at 95°C for 3 min with 1.5% dithiothreitol before application to a 7.5% polyacrylamide gel. The resulting gel was stained by silver staining kit (Pharmacia). Molecular weight markers were used for protein markers (Pharmacia) with phosphorylase-b (94 kDa), albumin (67 kDa), ovalbumin (43 kDa), carbonic anhydrase (30 kDa), trypsin inhibitor (20 kDa) and α-lactalbumin (14 kDa).

### Amino acid analysis

After SDS-PAGE under reducing conditions, the protein bands were transferred to a polyvinylidene difluoride membrane. The amino (N)-terminal amino acid sequence of the 70-kDa protein band that corresponded to the biological activity was analysed with a gas-phase protein sequencer (PPSQ-10; Shimadzu, Kyoto, Japan). Its amino acid sequence analysis was performed by Tokyo Research Laboratories, Kyowa Hakko Kogyo (Tokyo, Japan).

### Antibody studies

For the antibody studies, the mouse anti-human transferrin monoclonal antibody (Cosmo Bio, Tokyo, Japan) was used. This antibody is a mouse IgG molecule that has been shown to bind human transferrin specifically. BC47 cells were plated at $1 \times 10^6$ cells in 5 ml of serum-free medium added at various concentrations of antibodies (5 μg $ml^{-1}$, 10 μg $ml^{-1}$ and 20 μg $ml^{-1}$) in 25-$cm^2$ tissue culture flask. The cells were incubated at 37°C in a humidified atmosphere of 5% carbon dioxide and 95% air. Twenty-four hours and 48 h later, bromodeoxyuridine was added at a final concentration of 30 μg $ml^{-1}$ per flask, and the cells were reincubated for another hours. After bromodeoxyuridine labelling, the cells were trypsinized, washed three times with phosphate-buffered saline, and DNA-synthesizing cells were determined by the DNA–bromodeoxyuridine double-staining method using flow cytometry as reported previously (Tachibana et al, 1991). The same experiment was made with various amounts (5 μg $ml^{-1}$, 15 μg $ml^{-1}$ and 30 μg $ml^{-1}$) of the mouse anti-human transferrin receptor monoclonal antibody (Oncogene Science, NY, USA). Also, we investigated the effect of anti-human transferrin antibody on the growth-stimulating activity in BC47-conditioned medium. BC47 cells were plated at $1 \times 10^4$ cells $cm^{-2}$ in serum-free medium supplemented with both fivefold-concentrated BC47-conditioned medium and anti-human transferrin antibody (20 μg $ml^{-1}$) in 25-$cm^2$ tissue culture flasks. As a control, anti-human IgG antibody was used. To eliminate the possibility that the anti-human transferrin antibody was toxic to the cells, the same experiment was performed with KU7 cells that could grow well in serum-free medium, independent of plating cell density. Growth curves were obtained as described above.

© Cancer Research Campaign 1997

**Figure 1** Growth of bladder carcinoma cell lines plated at 3 ×$10^4$ cells $cm^{-2}$ in serum-free medium. Cell counting was performed by the trypan blue dye exclusion method. All studies were performed in duplicate with the mean values represented. The symbols indicate cell lines from: ○, BC47; ■, KU1; ▲, NBT; □, T24; and △, KU7

**Figure 2** Growth of BC47 and KU7 cells plated at $10^4$ cells $cm^{-2}$ in serum-free medium. Supplementation of BC47-conditioned medium made it possible for BC47 cells to grow at a low density. All studies were performed in duplicate with the mean values represented. The symbols indicate cell lines from: ○, BC47 added to 10% conditioned medium; ●, BC47; and △, KU7

### RESULTS

### Growth of bladder carcinoma cell lines in serum-free medium

Five bladder carcinoma cell lines were cultured in serum-free medium (Figure 1). As demonstrated by cell counts after a 24 h incubation period, the plating efficiency of three cell lines, with the exception of KU1 and BC47, were considerably reduced in serum-free medium. KU1 cells could survive without proliferation until 48 h, however, it could not be cultivated thereafter. On the other hand, BC47 cells grew well when plated at a density of 3 × $10^4$ cells $cm^{-2}$, but this cell line did not grow when plated at a lower density (Figure 2). Thus, it seems likely that the growth of BC47 cells in this serum-free medium was dependent on the plating cell density. Conversely, KU7 cells demonstrated vigorous growth in serum-free medium, regardless of plating cell density. T24 cells survived without proliferation during an experiment, while NBT cells could not be cultivated in serum-free medium. Fivefold-concentrated BC47-conditioned medium was found to stimulate the proliferation of BC47 cells plated in serum-free medium at 1 × $10^4$ cells $cm^{-2}$ (Figure 2). However, conditioned media from the other human bladder carcinoma cell lines NBT, T24 and KU1 did not exhibit a growth-stimulating activity on BC47 cell comparable to the effect of conditioned medium collected from BC47 (Figure 3). Moreover, when compared with control cultures, a significant decrease in cell numbers was observed by changing the medium at 24-h intervals during incubation for a period of 72 h (Table 1). These experiments suggest that growth-stimulating activity of conditioned medium collected from BC47 is not derived from a pre-existing substance in serum-free medium but is secreted by only BC47 cells growing in serum-free culture.

**Table 1** Effect of medium change on the growth of BC47 cells in serum-free medium

| | No. of cells per flask after a 72-h incubation period (× $10^6$ cells) |
|---|---|
| Control | 2.42 ± 0.10 |
| Sham treatment | 2.19 ± 0.08* |
| Medium change | 1.80 ± 0.21** |

BC47 cells were plated at 3 × $10^4$ cells $cm^{-2}$ tissue culture flasks. The medium was replaced with fresh medium at 24-h intervals. Sham treatment by removing the medium and then returning it was performed at 24-h intervals. All studies were performed in duplicate. Number of cells were expressed as mean ± s.d. *Not significant; ** $P < 0.01$ compared with the control.

**Figure 3** Effect of conditioned media collected from other bladder carcinoma cell lines on growth of BC47 cells in serum-free medium. BC47 cells were plated at a density of $10^4$ cells $cm^{-2}$ in 96-well plates. Twenty-five microlitres of conditioned medium of the various cell lines was added to each well. Cells were pulsed with 0.5 $\mu C_i$ [$^3$H]thymidine for 6 h. The results (■) are expressed as a percentage of c.p.m. obtained in control (□) and represent the mean values in triplicate

### Partial purification of growth-stimulating activity produced by BC47 cells

When concentrated BC47-conditioned medium was separated by gel filtration, the peak of growth-stimulating activity was observed in the high-molecular-weight fraction (Figure 4). In the anion-exchange chromatography, growth-stimulating activity was bound to the column at pH 8.6 and was eluted from the column by approximately 0.26 M sodium chloride (Figure 5). The lower-molecular-weight fractions with a minor activity by gel filtration were also

© Cancer Research Campaign 1997

**Figure 4** Gel filtration of BC47-conditioned medium. Two hundred microlitres of 50-fold-concentrated conditioned medium was applied to a 1.0x30-cm Superose 12 precolumn equilibrated with 0.05 M Tris-HCl (pH 8.6). Fractions (2 ml) were collected and their stimulation of [$^3$H]thymidine incorporation by BC47 cells was determined. The results are expressed as a percentage of c.p.m. obtained in control and represent the mean values in triplicate. The arrow indicates the peak relevant to the growth-stimulating activity. —, Absorbance at 280 nm; O–O, [$^3$H]thymidine incorporation analysed in the same manner; however, no growth-stimulating activity could be detected. The biologically active fraction obtained from gel filtration was applied to SDS-PAGE. As demonstrated in Figure 6, a light band at 70 kDa, which was close to the major dark band, was seen. When the active fraction from anion-exchange chromatography was also applied to SDS-PAGE, this 70-kDa band comigrated with the dark band. However, the 70-kDa band was considered to correspond to growth-stimulating activity because of an increase in its staining intensity according to purification.

### Structural analysis of the purified 70-kDa protein

In order to examine the relationship between the purified 70-kDa protein and other proteins, its N-terminal amino acid sequence was analysed with an automated protein sequencer. The N-terminal sequence up to the 17th amino acid residue was determined to be S-A-G-W-N-I-P-I-G-L-L-Y-X-D-L-P-E-, in which X seemed to be C (Cys). As a result of an investigation of the homology using the protein database, this sequence was found to be completely identical to the sequence of amino acid residues 125–141 from the N-terminus of human transferrin (MacGillivray et al, 1982; Yang et al; 1984).

### Inhibition of transferrin-like growth-stimulating activity by antibodies against human transferrin and transferrin receptor

To investigate the possibility of an autocrine mechanism in this system, monoclonal antibodies against human transferrin and transferrin receptor were tested for their effect on the proliferation of BC47 cells. Figure 7 showed that a decrease in the proportion of cells in S-phase was seen in a dose-dependent fashion by adding the anti-human transferrin antibody. Also, as shown in Figure 8, the decrease in the number of cells in S-phase of the cell cycle was manifested by adding the anti-human transferrin receptor antibody. As demonstrated previously, BC47-conditioned medium stimulated the proliferation of BC47 cells plated at 1 × $10^4$ cells

**Figure 5** Anion exchange chromatography of the high-molecular-weight fraction from gel filtration. The active fraction was applied to a 1.6x10-cm HiLoad Q Sepharose HP precolumn equilibrated with 0.05 M Tris-HCl (pH 8.6). Bound materials were eluted with a linear 0–0.5 M sodium chloride gradient in the same buffer. Fractions (5 ml) were collected, and their stimulation of [$^3$H]thymidine incorporation by BC47 cells was evaluated. The results are expressed as a percentage of c.p.m. obtained in control and represent the mean values in triplicate. The arrow indicates the peak relevant to the growth-stimulating activity. —, Absorbance at 280 nm; O–O [$^3$H]thymidine incorporation

**Figure 6** SDS-PAGE of purified active fractions from conditioned medium by BC47 cells. Samples were run on 7.5% polyacrylamide gel under reducing conditions. The lanes indicate samples from: (**A**) fraction 2 on gel filtration, (**B**) fraction 3 (the most active fraction) on gel filtration, (**C**) fraction 4 on gel filtration and (**D**) the most active fraction on anion exchange chromatography. The arrows to the left of the figure indicate the molecular weight markers in kDa. The arrow to the right of the figure indicates the growth-stimulating activity

$cm^{-2}$ in serum-free medium. However, the stimulatory effect of BC47-conditioned medium was largely blocked by adding the anti-human transferrin antibody, while KU7 cells grew well in the same experiment. Also, anti-human IgG antibody had no effect on the growth of BC47 in the serum-free medium (Figure 9).

### DISCUSSION

Serum has been used in nutritive media to supply to cultured cells a growth-stimulating activity with a complex, undefined and variable nature. The substitution of serum by standard media supplemented with nutrients and hormones of known composition and concentration simplifies the investigation of all classes of growth factors. Growth factors play a central role in cell transformation (Heldin and Westermark, 1984). In general, as tumours progress to a more malignant phenotype, they become less dependent on serum-derived growth factors for their growth in vitro and begin producing polypeptide growth factors (Rodeck and Herlyn, 1991), suggesting that autocrine growth mechanisms may be involved in malignant transformation. In several cell lines, it has been reported that the production of autocrine growth factors does occur. It has been shown that tumour cell growth can be regulated by normal growth factors, and the products of many viral and cellular oncogenes have been found to be related to growth factors or growth factor receptors (Wong and Passaro, 1989).

The growth of BC47 cells in serum-free medium demonstrated considerable dependence upon a high cell density. This was presumably not only because of technical factors such as the reduced plating efficiency but also because of an inferent density-dependence of the growth of BC47 cells in serum-free medium. Also, the number of proliferating BC47 cells decreased by periodic medium exchange. This phenomenon has been explained by the production of molecules that have a mitogenic effect and are transmitted between neighbouring cells. Cell proliferation apparently occurs when these substances reach a sufficient concentration to be stimulatory when bound to the target cells. The supernatant of growing cell cultures is therefore likely to contain not only growth-promoting factors (Cross and Dexter, 1991) but also various secretory factors (Liotta et al, 1986; Koshikawa et al, 1992; Akaogi et al, 1994). The present study also showed that culture of BC47 cells in serum-free conditions provided a suitable system for investigating such growth factors. We have shown that BC47 cells are able to secrete a 70-kDa molecule, which has growth-stimulating activity on BC47 cells themselves, into the chemically defined synthetic medium without transferrin. In addition, the N-terminal amino acid sequence of this molecule is homologous to the sequence of amino acid residues 125–141 of human transferrin, except for lacking amino acid residues 1–124 from N-terminus. BC47 is a rat bladder carcinoma cell line; however, strong homology is claimed to exist between the amino acid sequence of human and non-human transferrins (Bowman et al, 1988). Consequently, the bioactivity of human and rat transferrin is thought to be very similar, and we used monoclonal antibody to human transferrin and transferrin receptor in the blocking experiment.

Transferrin, the major iron-transporting protein in plasma, is a glycoprotein with a molecular weight of 80 kDa (MacGillivray et al, 1982). It transports ferric iron from the intestine, reticulo-endothelial system and liver parenchymal cells to all proliferating cells in the body. Previous experiments strongly indicate that the only function of transferrin in supporting cell proliferation is supplying cells with iron. Numerous in vitro studies have demonstrated the requirement of transferrin for proliferation and/or DNA synthesis, and thus transferrin has been included among the factors required for cell growth in serum-free media (Barnes and Sato, 1980). As discussed on previous reports (Shewale and Brew, 1982; MacGillivray et al, 1983), chemical and physical evidence indicates that the ferric iron bound at each site in transferrin is liganded with 1 or 2 histidyl residues, 2 or 3 tyrosines and a bicarbonate ion that probably interacts with an arginyl side chain. In the case of metal-binding protein and enzymes, in which the locations of binding site residues have been determined, two metal-liganding residues have been found to be no more than four residues apart in the amino acid sequence (Liljas and Rossman, 1974), presumably to form an initial locus for weak association before the formation of the complete binding site by conformational adjustments. On this basis, the only two candidates for components of the binding sites that are sufficiently close in the sequence are considered to be tyrosines 185 and 188 in the N-terminal domain and 514 and 517 in the carboxy-terminal domain of transferrin (Schwale and Brew, 1982). The 'transferin-like' molecule in serum-free medium conditioned by BC47 cells is likely to contain the above sequence of amino acids; therefore, this molecule is considered to act as an iron-carrier protein similar to native transferrin.

Another line of investigation revealed that transferrin may serve a role as a growth factor, independent of its function as a transporter of iron (May and Cuatrecasas, 1985). Transferrin is considered to be an

© Cancer Research Campaign 1997

**Figure 7** DNA and bromodeoxyuridine (BrdU) two-colour analysis of BC47 cells with anti-transferrin antibody by flow cytometer. Bivariate bromodeoxyuridine/DNA (green/red) fluorescence distributions are displayed in a dot-density graph with DNA distribution on axis of abscissa and bromodeoxyuridine fluorescence on axis of ordinate. BC47 cells were plated at $1 \times 10^6$ cells in 5 ml of medium in 25-$cm^2$ tissue culture flasks and various concentrations of antibodies were added: (**A**) none, (**B**) 5 $\mu g\ ml^{-1}$, (**C**) 10 $\mu g\ ml^{-1}$, (**D**) 20 $\mu g\ ml^{-1}$. The figures in each dot graph indicate the percentage of cells with positive staining

© Cancer Research Campaign 1997

**Figure 8** DNA and bromodeoxyuridine (BrdU) two-colour analysis of BC47 cells with anti-transferrin receptor antibody by flow cytometer. Bivariate bromodeoxyuridine/DNA (green/red) fluorescence distributions are displayed in the same dot-density graph as Figure 7. BC47 cells were plated at $1 \times 10^6$ cells in 5 ml of medium in 25-cm² tissue culture flasks. Various amounts of antibodies were added. (A) None, (B) 5 μg $ml^{-1}$, (C) 15 μg $ml^{-1}$, (D) 30 μg $ml^{-1}$. The figures in each dot graph indicate the percentage of cells with positive staining

 © *Cancer Research Campaign 1997*

**Figure 9** Effect of anti-transferrin antibody on growth-stimulating activity in BC47-conditioned medium. BC47 cells and KU7 cells were plated at $10^4$ cells $cm^{-2}$ in serum-free medium. At indicated days, the viable cells were counted and the mean values of duplicate experiments were represented. The symbols indicate cell lines from: ○, BC47 supplemented with 10% conditioned medium; □, BC47 supplemented with 10% conditioned medium and anti-transferrin antibody (20 µg $ml^{-1}$); △, BC47 supplemented with anti-human IgG; and ■, KU7 supplemented with anti-transferrin antibody (20 µg $ml^{-1}$)

autocrine regulator of cell proliferation in malignant tumour cells, where it may also be largely responsible for acquisition of serum independence (Vostrejs et al, 1988; Shapiro and Wagner, 1989). Growth factors exert their mitogenic effect by interaction with specific cell surface receptors on responsive cells. A prerequisite for autocrine growth stimulation is that the same cell both produces a growth factor and expresses the corresponding receptor. There is much evidence that tumour cell lines grown in vitro have the capacity to produce multiple growth factors that can act on the tumour cells themselves. Furthermore, tumour cell lines have provided examples of autocrine-stimulated cells for which, it is claimed, antibody to the growth factor can prevent cellular proliferation.

In the present study, it was shown that in transferrin-free serum-free medium BC47 cells exhibited growth that was dependent on the number of cells seeded, that the number of growing cells decreased as a result of regularly scheduled medium exchange and that, when conditioned medium was added to seeded numbers of BC47 cells that were incapable of growing, they became able to grow. It was also demonstrated that the amino acid sequence of the protein in the BC47-conditioned medium that showed growth-stimulating activity resembled that of transferrin, that anti-transferrin monoclonal antibody seemed to specifically block transferrin and the number of BC47 cells in the S-phase decreased concentration-dependently with this anti-transferrin monoclonal antibody, and that BC47 cell growth itself was inhibited by anti-transferrin monoclonal antibody. All of these findings strongly suggest that a 'transferrin-like' substance is a prominent component of the growth-stimulating activity produced by BC47 cells growing in serum-free medium, although immunoprecipitation of the conditioned medium and cell lysate is necessary to directly prove that BC47 cells produce a 'transferrin-like' substance. Expression of the transferrin receptor varied according to the cells, it was hardly detected at all in most somatic cells, which were in the resting phase. High concentrations are said to be present in cells that are actively growing, such as cancer cells and placental cells (Trowbridge and Omary, 1981), however, and we confirmed it to be present in BC47 cells as well. Furthermore, the monoclonal antibody to transferrin receptor is known to have very high specificity. Thus, the facts that, in the experiment in which monoclonal antibody to transferrin receptor was used, the number of S-phase cells decreased in a concentration-dependent manner, that no cytotoxic effect of the monoclonal antibodies themselves was observed, that no transferrin was contained in the serum-free medium and that high homology was found in the amino acid sequence of the 'transferrin-like' substance and transferrin strongly suggest that the 'transferrin-like' substance expresses its growth promoting action through transferrin receptors. These results also show that this 'transferrin-like' substance may act in 'public' or through an extracellular autocrine mechanism (Browder et al, 1989; Lang and Burgess, 1990). Specialized cellular proliferation may be limited by insufficient delivery of transferrin-bound iron from plasma during situations when iron is being withheld from the circulation. To evade the difficulty of obtaining iron, certain malignant cells have the ability to produce the 'transferrin-like' substance (Kitada and Hays, 1985; Morrone et al, 1988; Dittman and Petrides, 1991; Grüber et al, 1993) as well as native serum transferrin (Vostrejs et al, 1988; Vandewalle et al, 1989; Ohkawa et al, 1990; Stackpole et al; 1995). It could be hypothesized, therefore, that the capability of a specific subset of human bladder carcinomas to synthesize transferrin and/or a 'transferrin-like' substance might provide a source of available iron to support localized proliferation of bladder carcinoma cells in vivo in areas not well vascularized. In addition, the additional identification of such autocrine growth factors as the 'transferrin-like' substance and/or transferrin could lead to new therapeutic strategies for the treatment of bladder carcinoma in the future.

### ACKNOWLEDGEMENTS

We thank emeritus Professor Hiroshi Tazaki for reviewing the manuscript and Mari Okamoto for her assistance in preparing cell lines.

### REFERENCES

Akaogi K, Okabe Y, Funahashi K, Yoshitake Y, Nishikawa K, Yasumitsu H, Umeda M and Miyazaki K (1994) Cell adhesion activity of a 30-kDa major secreted protein from human bladder carcinoma cells. *Biochem Biophys Res Commun* **198**: 1046–1053

Barnes D and Sato G (1980) Serum-free cell culture: a unifying approach. *Cell* **22**: 649–655

Bowman BH, Yang F and Adrian GS (1988) Transferrin: evolution and genetic regulation of expression. In *Advances in Genetics*, Caspari EW. (ed.), pp. 1–38. Academic Press: San Diego

Browder TM, Dunbar CE and Nienhuis AW (1989) Private and public autocrine loops in neoplastic cells. *Cancer Cells* **1**: 9–17

Cross M and Dexter TM (1991) Growth factors in development, transformation, and tumorigenesis. *Cell* **64**: 271–280

Dittman KH and Petrides PE (1991) A 41 kDa transferrin related molecule acts as an autocrine growth factor for HL-60 cells. *Biochem Biophys Res Commun* **176**: 473–478

Grüber A, Pflüger KH, Schöneberger J, Wenzel E and Havemann K (1993) Biochemical characterization of a novel autocrine transferrin-like growth factor in acute myeloblastic leukemia. *Leuk Lymphoma* **11**: 435–441

Heldin CH and Westermark B (1984) Growth factors: mechanism of action and relation to oncogenes. *Cell* **37**: 9–20

Kitada S and Hays EF (1985) Transferrin-like activity produced by murine malignant T-lymphoma cell lines. *Cancer Res* **45**: 3537–3540

Koshikawa N, Yasumitsu H, Umeda M and Miyazaki K (1992) Multiple secretion of matrix serine proteinases by human gastric carcinoma cell lines. *Cancer Res* **52**: 5046–5053

Laemmli UK (1970) Cleavage of structural proteins during the assembly of the head of bacteriophage T4. *Nature* **227**: 680–685

Lang RA and Burgess AW (1990) Autocrine growth factors and tumourigenic transformation. *Immunol Today* **11**: 244–249

Liljas A and Rossmann MG (1974) X-ray studies of protein interactions. *Annu Rev Biochem* **43**: 475–507

Liotta LA, Mandler R, Murano G, Katz DA, Gordon RK, Chiang PK and Schiffmann E (1986) Tumor cell autocrine motility factor. *Proc Natl Acad Sci USA* **83**: 3302–3306

MacGillivray RTA, Mendez E, Sinha SK, Sutton MR, Lineback-Zins J and Brew K (1982) The complete amino acid sequence of human serum transferrin. *Proc Natl Acad Sci USA* **79**: 2504–2508

MacGillivray RTA, Mendez E, Shewale JG, Sinha SK, Lineback-Zins J and Brew K (1983) The primary structure of human serum transferrin. *J Biol Chem* **258**: 3543–3553

Masuda Y, Yoshitake Y and Nishikawa K (1988) Growth control of A431 cells in protein-free medium; secretory products do not affect cell growth. *In Vitro Cell Devel Biol* **24**: 893–898

Matsuda H, Matsumoto M, Haraguchi S and Kanai K (1989) Partial purification of a growth factor synthesized by a rat hepatoma cell line established in serum-free medium. *Cancer Res* **49**: 2118–2122

May WS Jr and Cuatrecasas P (1985) Transferrin receptor: its biological significance. *J Membr Biol* **88**: 205–215

Messing EM, Fahey JL, DeKernion JB, Bhuta SM and Bubbers JE (1982) Serum-free medium for in vitro growth of normal and malignant urinary bladder epithelial cells. *Cancer Res* **42**: 2392–2397

Messing EM, Bubbers JE, DeKernion JB and Fahey JL (1984) Growth stimulating activity produced by human bladder cancer cells. *J Urol* **132**: 1230–1234

Morrone G, Corbo L, Turco MC, Pizzano R, De Felice M, Bridges S and Venuta S (1988) Transferrin-like autocrine growth factor, derived from T-lymphoma cells, that inhibits normal T-cell proliferation. *Cancer Res* **48**: 3425–3429

Ohkawa K, Takada K, Takizawa N, Hatano T, Tsukada Y and Matsuda M (1990) Clear cell carcinoma of the human ovary synthesizes and secretes a transferrin with microheterogeneity of lectin affinity. *Febs Lett* **270**: 19–23

Rodeck U and Herlyn M (1991) Growth factors in melanoma. *Cancer Metastasis Rev* **10**: 89–101

Shapiro LE and Wagner N (1989) Transferrin is an autocrine growth factor secreted by Reuber H-35 cells in serum-free culture. *In Vitro Cell Dev Biol* **25**: 650–654

Shewale JG and Brew K (1982) Effects of $Fe^{3+}$ binding on the microenvironments of individual amino groups in human serum transferrin as determined by differential kinetic labeling. *J Biol Chem* **257**: 9406–9415

Sporn MB and Todaro GJ (1980) Autocrine secretion and malignant transformation of cell. *N Engl J Med* **303**: 878–880

Sporn MB and Roberts AB (1985) Autocrine growth factors and cancer. *Nature* **313**: 745–747

Stackpole CW, Kalbag SS and Groszek L (1995) Acquisition of in vitro growth autonomy during B16 melanoma malignant progression is associated with autocrine stimulation by transferrin and fibronectin. *In Vitro Cell Dev Biol* **31**: 244–251

Tachibana M (1982) Studies on cellular adhesiveness in five different culture cell lines derived from carcinoma of the urinary bladder. *Keio J Med* **31**: 127–148

Tachibana M, Deguchi N, Jitsukawa S, Baba S, Hata M and Tazaki H (1991) Quantification of cell kinetic characteristics using flow cytometric measurements of deoxyribonucleic acid and bromodeoxyuridine for bladder carcinoma. *J Urol* **145**: 963–967

Tachibana M, Miyakawa A and Tazaki H (1995) Autocrine growth of transitional cell carcinoma of bladder induced by granulocyte-stimulating factor. *Cancer Res* **55**: 3438–3443

Trowbridge IS and Omary MB (1981) Human cell surface glycoprotein related to cell proliferation is the receptor for transferrin. *Proc Natl Acad Sci USA* **78**: 3039–3043

Vandewalle B, Hornez L, Revillion F and Lefebvre J (1989) Secretion of transferrin by human breast cancer cells. *Biochem Biophys Res Commun* **163**: 149–154

Vostrejs M, Moran PL and Seligman PA (1988) Transferrin synthesis by small cell lung cancer cells acts as an autocrine regulator of cellular proliferation. *J Clin Invest* **82**: 331–339

Wong RS and Passaro E Jr (1989) Growth factors, oncogenes, and the autocrine hypothesis. *Surg Gynecol Obstet* **168**: 468–473

Yang F, Lum JB, McGill JR, Moore CM, Naylor SL, van Bragt PH, Baldwin WD and Bowman BH (1984) Human transferrin: cDNA characterization and chromosomal localization. *Proc Natl Acad Sci USA* **81**: 2752–2756

© Cancer Research Campaign 1997